# Draft Genome Sequence of Xanthobacter aminoxidans ATCC BAA-299^T^

**DOI:** 10.1128/mra.00548-22

**Published:** 2022-07-13

**Authors:** Fardeen A. Siddiqui, Shoshana Trudel, Abigail E. Goen, Syeda A. Fatima, Kyle S. MacLea

**Affiliations:** a Biotechnology Program, University of New Hampshire, Manchester, New Hampshire, USA; b Biology Program, University of New Hampshire, Manchester, New Hampshire, USA; c Department of Life Sciences, University of New Hampshire, Manchester, New Hampshire, USA; Wellesley College

## Abstract

Xanthobacter aminoxidans is a Gram-negative pleomorphic and diazotrophic Knallgas bacillus that undergoes asymmetric budding of V-shaped branched cells during cell division. Like other *Xanthobacter* spp., cells are yellow from production of zeaxanthine dirhamnoside. We sequenced strain 14a^T^ (= ATCC BAA-299^T^) and report a genome size of 5,829,486 bp with a G+C content of 67.9%.

## ANNOUNCEMENT

The family *Xanthobacteraceae* of the class *Alphaproteobacteria*, which is found in freshwater, wetlands, soils, marine sediments, and plant roots and in waste treatment systems and polluted sites, contains aerobic chemoheterotrophs, although facultative chemolithoautotrophy utilizing hydrogen is also commonly found (1–13). Xanthobacter aminoxidans (strain 14a^T^ = VKM B-2254^T^ = ATCC BAA-299^T^) was isolated from the activated sludge of a sewage purification system at the Baikal paper mill in Russia in 1979 (8). X. aminoxidans cells are Gram-negative pleomorphic rods that branch into V-shaped cells in asymmetric cell division (8, 10). Xanthobacter aminoxidans has varied metabolic capabilities, including growing autotrophically in H_2_ plus O_2_ plus CO_2_, reducing nitrates to nitrites, and utilizing many carbon sources, including glutamine and methanol; the latter function is potentially useful to combat methanol pollution in wastewater treatment (10, 12, 14). Additionally, X. aminoxidans, like other *Xanthobacter* species, is capable of fixing N_2_ under low-oxygen conditions (10, 12). While some *Xanthobacter* strains have been sequenced (12, 15–17), X. aminoxidans was still not sequenced, which led to the sequencing effort described below.

Xanthobacter aminoxidans ATCC BAA-299^T^ was obtained from ATCC (Manassas, VA, USA) in lyophilized form. Bacteria from an isolated colony were cultured in tryptic soy broth for 24 h at 30°C. A QIAamp DNA Mini Kit (Qiagen, Valencia, CA, USA) was employed to extract non-size-selected genomic DNA (gDNA), and the KAPA HyperPlus kit (KR1145, v.5.19 [KK8515]; Kapa Biosystems, Wilmington, MA, USA) was then used to create the sequencing library by enzymatic fragmentation with HyperPlus end repair. The DNA library was sequenced on an Illumina HiSeq 2500 instrument by the Hubbard Center for Genome Studies at the University of New Hampshire (Durham, NH, USA), generating 250-bp paired-end fragments. The resulting reads were trimmed by Trimmomatic v.0.38 (settings: paired-end mode with a window size of 4, quality requirement of 15, and minimum read length of 36) (18). SPAdes v.3.13.0 (19) assembled 23,117,624 trimmed short reads with default bacterial parameters. After removal of small (<500 bp) and low-coverage (<65×) contigs, gene prediction and annotation for the remaining 78 X. aminoxidans contigs were completed using the NCBI Prokaryotic Genome Annotation Pipeline (PGAP) v.6.1 (settings: best-placed reference protein set and GeneMarkS-2+) (20). The largest contig was 596,261 bp long, with a genome *N*_50_ value of 242,177 bp. The total genome length was 5,829,267 bp, with a G+C content of 67.9%, similar to other members of the genus, such as Xanthobacter oligotrophicus 29k^T^ (5,313,426 bp, with a G+C content of 67.9%) (12). A total of 5,542 total genes were identified, of which 5,415 were protein-coding genes. The genome contained 54 RNA genes (1 complete copy of each rRNA, 47 tRNAs, and 4 noncoding RNAs) and 73 pseudogenes. The genome assembly was estimated to contain 100% of the expected highly conserved essential genes by benchmarking universal single-copy orthologs (BUSCO) v.5.2.2 analysis (default bacterial lineage settings, with no duplicated BUSCOs detected) (21–23), with an average genome coverage of 1,156×.

Consistent with known metabolism, we found by RAST analysis (24) genetic signatures of diverse types of nitrogen metabolism (from N_2_ fixation to ammonia assimilation), carbohydrate metabolism (including C1 and C2 metabolic functions), and catabolism of diverse aromatic compounds. Interestingly, we also found a number of phage/prophage elements within the genome sequence, as well as 17 flagellar genes, although X. aminoxidans is described as a nonmotile species (8, 10).

### Data availability.

The Xanthobacter aminoxidans ATCC BAA-299^T^ whole-genome sequencing (WGS) project was deposited in DDBJ/ENA/GenBank under accession number JAMJXC000000000. The raw data from BioProject accession number PRJNA509625 were submitted to the NCBI Sequence Read Archive (SRA) under accession number SRX15392594.
